# Autopsy-Confirmed Non-Paraneoplastic Lambert–Eaton Myasthenic Syndrome with Cerebellar Degeneration: A Case Report

**DOI:** 10.3390/diagnostics16132124

**Published:** 2026-07-07

**Authors:** Hajime Iwata, Jun Ikezawa, Masayuki Honda, Ryo Morishima, Yuta Amagasaki, Tomonari Seki, Takahiro Kiriu, Keisuke Ishizawa, Kazushi Takahashi, Haruka Okada

**Affiliations:** 1 Department of Pathology, Tokyo Metropolitan Tama Medical Center, Fuchu 183-8524, Japan; kirinama@mercury.sannet.ne.jp (T.K.); haruka_okada@tmhp.jp (H.O.); 2 Department of Neurology, Tokyo Metropolitan Neurological Hospital, Fuchu 183-0042, Japan; jun_ikezawa@tmhp.jp (J.I.); masayuki_honda@tmhp.jp (M.H.); riyou_morishima@tmhp.jp (R.M.); kazushi_takahashi@tmhp.jp (K.T.); 3 Department of Emergency and General Medicine, Tokyo Metropolitan Tama Medical Center, Fuchu 183-8524, Japan; yuta_amagasaki@tmhp.jp; 4 Department of Neurology, Tokyo Teishin Hospital, Chiyoda-ku, Tokyo 102-8798, Japan; tomseki@tth-japanpost.jp; 5 Department of Laboratory Medicine (Neuropathology), Tokyo Metropolitan Neurological Hospital, Fuchu 183-0042, Japan; ishizawa@saitama-med.ac.jp; 6 Department of Pathology, Saitama Medical University, Saitama 350-0495, Japan

**Keywords:** Lambert–Eaton myasthenic syndrome, cerebellar degeneration, Purkinje cell loss, voltage-gated calcium channel, autopsy, non-paraneoplastic, antibody-mediated, cerebellar ataxia

## Abstract

**Background and Clinical Significance:** Lambert–Eaton myasthenic syndrome (LEMS) is mediated by antibodies against P/Q-type voltage-gated calcium channels (VGCCs) and is classified as paraneoplastic (T-LEMS) or non-paraneoplastic (NT-LEMS). Cerebellar degeneration is recognized in T-LEMS, but pathological confirmation in NT-LEMS has not been reported. **Case Presentation:** A 79-year-old man developed progressive ataxic gait and dysarthria at age 76 and was diagnosed with LEMS based on repetitive nerve stimulation findings and anti-P/Q-type VGCC antibodies. No malignancy was identified during more than 40 months of surveillance, and comprehensive autopsy revealed no occult tumor. After hospitalization for erythroderma and pneumonia, he died of respiratory failure. Postmortem examination revealed severe Purkinje cell loss with Bergmann gliosis in the anterior lobe and tuber vermis, accompanied by torpedoes and empty baskets, without significant inflammation. These findings indicate that NT-LEMS can reach the same VGCC-associated Purkinje cell endpoint previously documented only in paraneoplastic LEMS, despite different upstream triggers. **Conclusions:** This first autopsy-confirmed case of NT-LEMS with cerebellar degeneration supports a shared, non-inflammatory VGCC-mediated pathway of Purkinje cell injury across LEMS subtypes.

## 1. Introduction

Lambert–Eaton myasthenic syndrome (LEMS) is an autoimmune disorder of the neuromuscular junction caused by antibodies against voltage-gated calcium channels (VGCCs), primarily the P/Q-type subunits; more than 85% of patients harbor these antibodies [1,2]. Approximately 50–60% of cases are paraneoplastic (T-LEMS), predominantly associated with small cell lung carcinoma (SCLC), while the remaining 40–50% are classified as non-paraneoplastic (NT-LEMS), in which no underlying malignancy has been identified [1]. LEMS is rare, with an estimated prevalence of approximately 2.7–4 per million [3,4]. The median age of onset is 51 years in NT-LEMS with a slight female predominance (54%), compared with 63 years and a male predominance in SCLC-LEMS [5]. Although life expectancy in NT-LEMS is comparable to the general population, physical health-related quality of life is significantly impaired [5].

P/Q-type VGCCs mediate neurotransmitter release at motor nerve terminals, constituting the molecular basis of neuromuscular junction impairment in LEMS [1]. These channels are also highly expressed in the cerebellar cortex, particularly in the molecular layer at parallel fiber–Purkinje cell synapses [6], providing the basis for dual targeting of the neuromuscular junction and cerebellum by VGCC antibodies. Cerebellar ataxia is a well-recognized neurological complication of LEMS, reported in 55–64% of T-LEMS compared with 19% in NT-LEMS [7], and can be a clinical manifestation of underlying cerebellar degeneration. Autopsy studies in T-LEMS have demonstrated Purkinje cell loss with Bergmann gliosis [6,8,9,10], and reduction of cerebellar P/Q-type VGCC density has been documented [6]. These findings raise the question of whether T-LEMS and NT-LEMS, despite different initiating immune triggers, can converge on a shared VGCC-associated cerebellar endpoint.

However, pathological confirmation of cerebellar involvement in NT-LEMS has never been reported. Clinical reports have described cerebellar ataxia in NT-LEMS [11,12,13], and a VGCC-antibody slice-culture study showed Purkinje cell binding followed by neuronal death [14]. These observations support biological plausibility but do not establish the autopsy pathology of NT-LEMS. Herein, we describe the clinicopathological findings of a patient with NT-LEMS who developed progressive cerebellar ataxia and subsequently died of pneumonia. Comprehensive postmortem examination confirmed Purkinje cell loss and excluded occult malignancy, allowing us to examine whether NT-LEMS can reach the same VGCC-associated Purkinje cell endpoint previously documented only in paraneoplastic LEMS.

## 2. Case Presentation

### 2.1. Clinical Course

A 79-year-old man with a history of type 2 diabetes mellitus and heavy smoking presented with progressive ataxic gait and dysarthria at age 76. He had no family history of neurological disorders and no history of alcohol use.

These symptoms began a few days after influenza vaccination, when he first developed unsteadiness and slurred speech. Over the following months, he experienced repeated backward falls and began using a walking cane. Neurological examination revealed dysarthria, truncal ataxia, and absent deep tendon reflexes in all extremities. Given his long-standing diabetes, diabetic neuropathy was initially considered a possible cause of areflexia; however, deep tendon reflexes became elicitable after brief exercise, suggesting a presynaptic neuromuscular junction disorder rather than peripheral neuropathy.

Laboratory investigations demonstrated positive anti-P/Q-type VGCC antibodies (96.3 pmol/L; normal < 20 pmol/L). The paraneoplastic antibody panel was negative for anti-Hu, anti-Yo, anti-Ri, anti-CV2, anti-amphiphysin, anti-PNMA2, anti-recoverin, anti-Tr (DNER), anti-Zic4, and anti-GAD65 antibodies; anti-SOX1 antibodies showed borderline reactivity (±) on a commercial line blot assay. Repetitive nerve stimulation showed post-exercise facilitation (170% increment of compound muscle action potential in the abductor pollicis brevis).

Whole-body FDG PET/CT revealed no evidence of malignancy, and subsequent tumor surveillance available at our institution also remained negative; liquid biopsy for circulating tumor DNA was not available. He was diagnosed with LEMS and possible paraneoplastic cerebellar degeneration.

**Figure 1:**
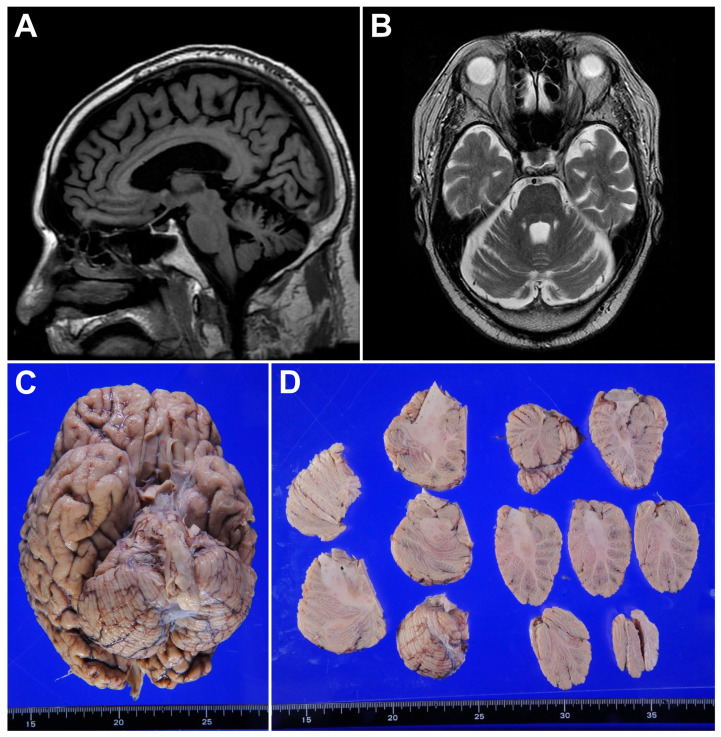
Neuroimaging and gross findings demonstrating mild cerebellar atrophy. (**A**) T1-weighted sagittal MRI. (**B**) T2-weighted axial MRI. (**C**) Basal view of the brain at autopsy. (**D**) Cut surface of the cerebellum; the right hemisphere was sectioned horizontally, and the left hemisphere was sectioned coronally.

Treatment included plasma exchange, immunoadsorption, and intravenous methylprednisolone pulse therapy. At approximately 22 months after symptom onset, methylprednisolone pulse therapy was initiated, resulting in improvement of his Scale for the Assessment and Rating of Ataxia (SARA) score from 19 to 13 [15]. Brain MRI at that time revealed mild cerebellar atrophy (Figure 1A,B). Oral prednisolone maintenance therapy was subsequently started (30 mg/day) and tapered to 22.5 mg/day over approximately 2.5 months. However, his ataxia gradually progressed despite continued immunotherapy, with SARA scores fluctuating between 13 and 20 over the following months. Since the patient had a long-standing history of type 2 diabetes requiring insulin therapy for approximately 20 years, glycemic control became increasingly difficult during steroid treatment.

At 35 months after symptom onset, he developed acute coronary syndrome and underwent percutaneous coronary intervention. Dual antiplatelet therapy with aspirin and clopidogrel was initiated, and aspirin was subsequently switched to rivaroxaban due to persistent atrial tachycardia, while clopidogrel was continued; catheter ablation was later performed for atrial fibrillation. Approximately seven weeks after starting rivaroxaban, scattered erythema appeared on the trunk and extremities. Over the following several weeks, the erythema progressively spread and coalesced, with desquamation developing throughout the body. He was ultimately diagnosed with erythroderma, with rivaroxaban suspected as a causative agent. Shortly thereafter, he developed left lower extremity pain and was unable to self-administer his medications, including prednisolone and insulin. He was admitted to our hospital for erythroderma and pneumonia. High-dose corticosteroid pulse therapy was administered for erythroderma. He subsequently developed diabetic ketoacidosis, attributed to the combination of interruption of insulin therapy due to inability to self-administer medications, high-dose corticosteroid pulse therapy for erythroderma, ongoing sodium–glucose cotransporter 2 inhibitor use, and metabolic stress from erythroderma and infection. Although metabolic acidosis was corrected, pneumonia progressed and he died of respiratory failure 4 days after admission. An autopsy including the brain was performed.

### 2.2. Postmortem Findings

The brain weighed 1309 g. The basal view showed no apparent mass lesion (Figure 1C). Grossly, mild cerebellar atrophy, particularly of the vermis, was observed on the cut surface (Figure 1D).

**Figure 2:**
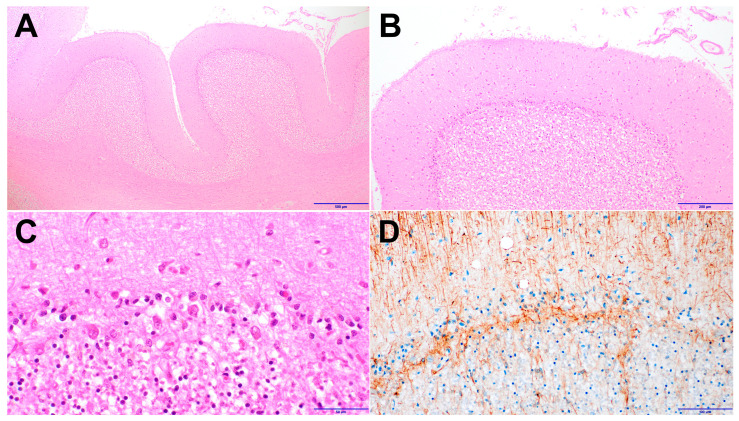
Purkinje cell loss and Bergmann gliosis in the cerebellar vermis. (**A**) Low-power view showing severe Purkinje cell loss (H&E, 40× magnification). (**B**) Purkinje cell loss with Bergmann gliosis (H&E, 100× magnification). (**C**) Bergmann gliosis (H&E, 400× magnification). (**D**) Bergmann glia proliferation (GFAP immunostaining, 200× magnification).

Histologically, Purkinje cell loss was observed in the cerebellar vermis involving both the anterior lobe (paleocerebellum) and the tuber vermis (neocerebellum). The cerebellar cortex showed severe loss of Purkinje cells (Figure 2A,B). Purkinje cell dropout was accompanied by Bergmann gliosis (Figure 2B,C). Bergmann glia proliferation was evident on glial fibrillary acidic protein (GFAP) immunostaining (Figure 2D).

**Figure 3:**
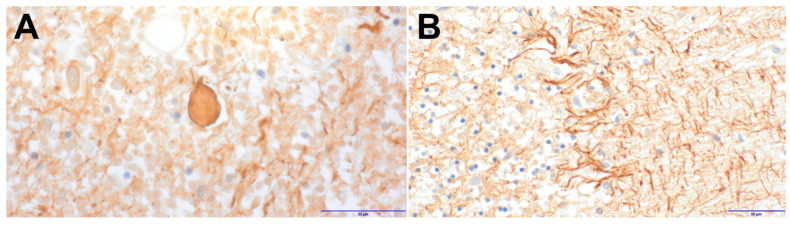
Axonal pathology in the cerebellar vermis. (**A**) Torpedo formation (axonal spheroid) in the granular layer (neurofilament protein immunostaining, 400× magnification). (**B**) Empty baskets demonstrating Purkinje cell loss (neurofilament protein immunostaining, 400× magnification).

Torpedo formation (axonal spheroids) was observed in the granular layer (Figure 3A). Empty baskets were evident on neurofilament protein immunostaining, confirming Purkinje cell loss (Figure 3B).

The dentate nucleus was preserved; no neuronal loss or grumose degeneration was detected on H&E or immunostaining for synaptophysin. Neither significant inflammatory cell infiltration nor prominent microglial activation was observed, suggesting a chronic degenerative process rather than an inflammatory process.

The cerebrum showed no significant changes in the gray matter, white matter, or basal ganglia, and α-synuclein immunostaining revealed no glial cytoplasmic inclusions. Notably, no neuronal loss was observed in the hippocampus, a region highly vulnerable to ischemia or hypoxia, suggesting that the Purkinje cell loss was unlikely to be of ischemic or hypoxic origin.

The lungs showed bilateral pneumonia with neutrophilic infiltration and fibrin deposition, along with pulmonary edema and congestion. The heart demonstrated chronic ischemic changes with myocardial fibrosis, which may have contributed to pulmonary congestion. Comprehensive autopsy examination, including extensive sampling of both lungs and systemic lymph nodes, revealed no evidence of SCLC or other malignancies.

The neuropathological diagnosis was cerebellar degeneration with Purkinje cell loss in the setting of LEMS, without evidence of underlying malignancy.

## 3. Discussion

To our knowledge, this is the first autopsy-confirmed case of NT-LEMS with cerebellar degeneration, providing the first neuropathological evidence that Purkinje cell loss in LEMS extends beyond paraneoplastic settings. Cerebellar ataxia is reported in 55–64% of T-LEMS but only 19% of NT-LEMS [7], and a systematic review of 67 patients with coexistent LEMS and cerebellar ataxia found that 28.4% had no detectable malignancy [13], indicating that cerebellar involvement is not exclusive to paraneoplastic forms. However, the autopsy-confirmed cases identified in the literature were all SCLC-associated (Table 1), and pathological confirmation in NT-LEMS had been lacking. The present case fills this gap by showing that NT-LEMS can converge on the same core pathological pattern, namely severe Purkinje cell loss with Bergmann gliosis and minimal inflammation, despite the absence of malignancy.

The main mimics were alcoholic cerebellar degeneration, the cerebellar type of multiple system atrophy (MSA-C), hereditary spinocerebellar ataxia, and terminal hypoxic–ischemic injury [16]. MSA-C was excluded by the absence of α-synuclein-positive glial cytoplasmic inclusions; hereditary spinocerebellar ataxia was considered unlikely given the absence of a family history; hypoxic–ischemic injury was excluded by the preservation of hippocampal neurons, which share the cerebellar cortex’s selective vulnerability to hypoxia [16]; and alcoholic cerebellar degeneration was not supported by the clinical history. These exclusions, together with electrophysiologically confirmed LEMS, anti-P/Q-type VGCC antibodies, chronic Purkinje cell loss with Bergmann gliosis, torpedoes, and empty baskets, and no occult malignancy at autopsy, supported autoimmune NT-LEMS-associated cerebellar degeneration. Although the symptoms began a few days after influenza vaccination, the 40-month progressive course and chronic autopsy pathology were not typical of a monophasic post-vaccination acute cerebellitis [17].

Occult malignancy remained the main diagnostic concern because of the heavy smoking history and borderline anti-SOX1 reactivity. Borderline line-blot SOX1 reactivity alone was insufficient to establish SCLC-associated LEMS [18]. Liquid biopsy was not performed; nevertheless, no tumor was detected during more than 40 months of surveillance or at comprehensive autopsy, supporting the diagnosis of NT-LEMS.

Compared with the previously reported SCLC-associated autopsy cases (disease duration 11–25 months; Table 1), the present NT-LEMS case followed a longer course of approximately 40 months and showed Purkinje cell loss involving both the anterior lobe and the tuber vermis. Immunotherapy produced only transient improvement, and the ataxia progressed despite continued treatment, with autopsy ultimately demonstrating severe Purkinje cell loss. The severity of this loss exceeded what the mild antemortem MRI and gross autopsy findings had suggested (Figure 1A–D), a clinicopathological gap likewise reported in anti-P/Q-type VGCC-associated paraneoplastic cerebellar degeneration, in which the cerebellar hemisphere appeared macroscopically unremarkable despite histological Purkinje cell loss [19].

Although T-LEMS and NT-LEMS share P/Q-type VGCCs as the principal antibody target, their upstream immune contexts differ. T-LEMS is linked to SCLC-driven antitumor immunity, whereas NT-LEMS lacks a tumor-derived trigger; reported non-tumor trigger clues include domain IV S5–S6 linker recognition and short-epitope sequence similarities of uncertain significance, with glucose-regulated protein 78 antibody-mediated blood–brain barrier modulation proposed as a possible access factor [20,21,22]. These differences concern trigger and antibody access rather than the downstream cerebellar lesion. They therefore do not preclude convergence on a common VGCC-associated endpoint.

The present autopsy supports such convergence at the effector level. Severe Purkinje cell loss with Bergmann gliosis formed the core lesion, with torpedoes and empty baskets as accompanying markers of chronic Purkinje-cell-centered degeneration [16,23,24,25]. Significant inflammation was absent. This cortical pattern overlaps with the previously reported autopsy-confirmed T-LEMS cases, in which Purkinje cell loss and cerebellar cortical gliosis were common findings (Table 1). The paucity of inflammation is also consistent with a VGCC-associated profile, because VGCC-associated paraneoplastic cerebellar degeneration shows less inflammatory pathology than Yo-associated disease [19]. Thus, despite different upstream immune contexts, NT-LEMS can reach the same core VGCC-associated, non-inflammatory Purkinje cell injury endpoint previously documented in T-LEMS.

## 4. Conclusions

This is the first autopsy-confirmed case of NT-LEMS with cerebellar degeneration. The findings show that Purkinje cell loss in LEMS is not restricted to paraneoplastic disease and can occur in the absence of malignancy. Together with prior paraneoplastic autopsy cases, this case supports a model in which different upstream immune triggers in T-LEMS and NT-LEMS converge on a shared P/Q-type VGCC-mediated, non-inflammatory pathway of Purkinje cell injury. By documenting this lesion in a tumor-free setting, the case provides a reference point for interpreting progressive ataxia in future NT-LEMS cases. Comprehensive autopsy was essential to document this pathological endpoint and exclude occult malignancy, thereby extending the pathological spectrum of LEMS-associated cerebellar degeneration beyond paraneoplastic disease.

## Figures and Tables

**Table 1:** Comparison of autopsy-confirmed LEMS with cerebellar degeneration cases.

Author/Year	Age/Sex	Tumor	Anti-P/Q-Type VGCC Ab	Initial Symptom	Purkinje Cell Status	Distribution	Duration
Satoyoshi 1973 [8]	60/M	SCLC	NA	Leg fatigue, ataxia	Lost	Neocerebellum-predominant	16 mo
Shirabe 1981 [9]	55/M	SCLC	NA	Fatigue, ataxia	Lost	Paleocerebellum	18 mo
Kobayashi 1988 [10]	37/M	SCLC	NA	Vertigo, ataxia	Lost	ND	25 mo
Fukuda 2003 [6] (Case 1)	79/M	SCLC (mixed)	Positive	Dizziness, ataxia	Lost	ND	11 mo
Fukuda 2003 [6] (Case 2)	69/M	SCLC	Positive	Gait disturbance	Lost	ND	18 mo
Fukuda 2003 [6] (Case 3)	74/F	SCLC	Positive	Ataxia, dysarthria	Lost	ND	11 mo
Present case	79/M	None	Positive	Ataxia, dysarthria	Lost	Anterior lobe, tuber vermis	40 mo

Abbreviations: SCLC, small cell lung carcinoma; VGCC, voltage-gated calcium channel; Ab, antibody; mo, months; NA, not assessed in the original report; ND, not described in the original report; M, Male; F, Female. Duration indicates time from neurological symptom onset to death.

## Data Availability

The data presented in this study are available on request from the corresponding author due to privacy restrictions.

## Abbreviations

The following abbreviations are used in this manuscript:

LEMS	Lambert–Eaton myasthenic syndrome
VGCC	Voltage-gated calcium channel
T-LEMS	Tumor-associated (paraneoplastic) LEMS
NT-LEMS	Non-paraneoplastic or idiopathic LEMS
SCLC	Small cell lung carcinoma
SARA	Scale for the Assessment and Rating of Ataxia
GFAP	Glial fibrillary acidic protein
